# Signal-transducing adapter protein-1 is required for maintenance of leukemic stem cells in CML

**DOI:** 10.1038/s41388-020-01387-9

**Published:** 2020-07-13

**Authors:** Jun Toda, Michiko Ichii, Kenji Oritani, Hirohiko Shibayama, Akira Tanimura, Hideaki Saito, Takafumi Yokota, Daisuke Motooka, Daisuke Okuzaki, Yuichi Kitai, Ryuta Muromoto, Jun-ichi Kashiwakura, Tadashi Matsuda, Naoki Hosen, Yuzuru Kanakura

**Affiliations:** 1 Department of Hematology and Oncology, Osaka University Graduate School of Medicine, Suita, Japan; 2 Department of Hematology, Graduate School of Medical Science, International University of Health and Welfare, Narita, Japan; 3 Genome Information Research Center, Research Institute for Microbial Diseases, Osaka University, Suita, Japan; 4 Department of Immunology, Graduate School of Pharmaceutical Sciences, Hokkaido University, Sapporo, Japan; 5 Sumitomo Hospital, Osaka, Japan

**Keywords:** Apoptosis, Apoptosis, Cancer stem cells, Cancer stem cells

## Abstract

The family of signal-transducing adapter proteins (STAPs) has been reported to be involved in a variety of intracellular signaling pathways and implicated as transcriptional factors. We previously cloned STAP-2 as a c-Fms interacting protein and explored its effects on chronic myeloid leukemia (CML) leukemogenesis. STAP-2 binds to BCR-ABL, upregulates BCR-ABL phosphorylation, and activates its downstream molecules. In this study, we evaluated the role of STAP-1, another member of the STAP family, in CML pathogenesis. We found that the expression of STAP-1 is aberrantly upregulated in CML stem cells (LSCs) in patients’ bone marrow. Using experimental model mice, deletion of STAP-1 prolonged the survival of CML mice with inducing apoptosis of LSCs. The impaired phosphorylation status of STAT5 by STAP-1 ablation leads to downregulation of antiapoptotic genes, Bcl-2 and Bcl-xL. Interestingly, transcriptome analyses indicated that STAP-1 affects several signaling pathways related to BCR-ABL, JAK2, and PPARγ. This adapter protein directly binds to not only BCR-ABL, but also STAT5 proteins, showing synergistic effects of STAP-1 inhibition and BCR-ABL or JAK2 tyrosine kinase inhibition. Our results identified STAP-1 as a regulator of CML LSCs and suggested it to be a potential therapeutic target for CML.

## Introduction

Chronic myeloid leukemia (CML) is a clonal myeloproliferative disorder that is caused by hematopoietic stem cells (HSCs) expressing the BCR-ABL fusion oncoprotein. This constitutively active tyrosine kinase influences multiple signal transduction pathways, such as Ras/Raf/mitogen-activated protein kinase (MAPK), Janus kinase (JAK)/signal transducer and activator of transcription (STAT), phosphatidylinositol 3-kinase (PI3K)/Akt, and nuclear factor-κB (NF-κB), leading to peripheral granulocytosis, splenomegaly, and thrombocytosis [1, 2]. Since the use of tyrosine kinase inhibitors (TKIs) for the management of CML was approved, its therapeutic outcomes have been dramatically improved. However, approximately 50% of patients relapse within 12 months after discontinuing TKI therapy, even if they have achieved a deep molecular remission [3–5]. Many patients require lifelong TKI treatment, which may lead to adverse reactions affecting the quality of life and escalation of costs of treatment.

These clinical observations indicated that the inhibition of BCR-ABL kinase activity alone cannot eliminate CML stem cells (LSCs) [6–8]. Previously, it has been reported that BCR-ABL-positive stem cells are detected in patients who maintain stable molecular response after long-term TKI treatment and even after stopping TKI therapy for many years [9–11]. Recent studies with single-cell molecular approach showed that a distinct subset of LSCs with deep quiescent signature persists during TKI-induced remission [12, 13]. These primitive CML LSCs do not depend on BCR-ABL1 activity for survival [14, 15]. Several key signaling molecules and pathways have been proposed to regulate survival and self-renewal of CML LSCs in the presence of TKI, including transforming growth factor (TGF)-β, Foxo, Hedgehog, Wnt, and JAK/STAT signaling [16–25]. The bone marrow (BM) microenvironment also sustains CML LSCs [26, 27]. Elucidation of molecular mechanisms that maintain LSCs is needed to develop novel therapeutic approaches for CML. However, the precise underlying mechanisms of BCR-ABL kinase inhibitor-resistance in LSC remain largely unknown.

The family of signal-transducing adapter proteins (STAPs), which contains STAP-1 and STAP-2, has been implicated in various intracellular signaling pathways. STAP-1 was cloned as a c-Kit interacting protein by yeast two-hybrid screening of an HSC library in 2000 [28]. Three years later, we cloned STAP-2 as a c-Fms interacting protein [29]. These proteins share 33% of overall amino acid identity and have pleckstrin homology and Src-homology 2 (SH2)-like domains, which represent typical structures of an adapter protein [28, 29]. STAPs have been shown to interact with several tyrosine phosphorylated proteins, including IκB kinase complex, STAT3 and STAT5, during inflammatory/immune response and tumorigenesis [28, 30–32]. Recently, we reported that STAP-2 binds to BCR-ABL via its SH2-like domain, and enhances proliferation of CML cells as well as their resistance to imatinib [32].

It is still unknown whether STAP-1 plays a role in CML development, although STAP-1 is expected to exhibit similar functions based on the structural homology between STAP-1 and STAP-2. Given the hematopoietic expression of STAP-1, we hypothesized that STAP-1 might also contribute to the function of LSCs. Here we investigated the role of STAPs in CML using an in vivo experimental mouse model.

## Results

### Aberrant upregulation of STAP-1 expression in human CML stem cells

**Fig. 1:**
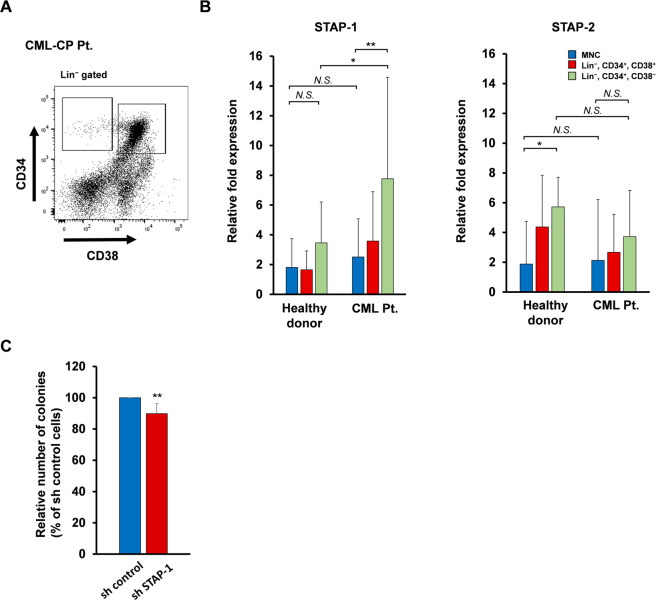
The expression of STAP-1 aberrantly increased in human CML LSC subset. **a**, **b** BM cells were collected from healthy donor (*n* = 7) and newly diagnosed chronic CML patients (*n* = 8), and Lin^−^ CD34^+^ CD38^−^ hematopoietic stem cells and Lin^−^ CD34^+^ CD38^+^ hematopoietic progenitor cells were sorted using flow cytometry. **a** The representative data of flow cytometry are shown. **b** STAP-1 and STAP-2 mRNA were measured by real-time RT-PCR in the indicated subsets. Bar graph shows expression levels of STAP-1 and STAP-2 mRNAs normalized to data from MNC fraction of healthy donor. **c** Lin^−^ CD34^+^ BM cells from newly diagnosed chronic CML patients (*n* = 6) were transduced with control or STAP-1 shRNA lentivirus, cultured in triplicates in methylcellulose medium for 14 days, and colonies were counted. Data are shown as mean ± SD. *p < 0.05 and **p < 0.01 indicate significant difference. MNC mononuclear cells, NS not significant.

We first evaluated STAP-1 and STAP-2 expression in human BM. We collected samples from healthy donors and CML chronic phase (CML-CP) patients, and isolated Lin^−^ CD34^+^ CD38^+^ hematopoietic progenitor cells and Lin^−^ CD34^+^ CD38^−^ HSCs (Fig. 1a). Interestingly, STAP-1 mRNA expression in the stem cell compartment was significantly higher in CML patients than that in healthy donors, whereas we found no difference in the expression of STAP-2 (Fig. 1b). Our observation of increased STAP-1 expression in CML stem cells corresponded to two gene expression databases obtained from previous studies [12, 33]. To study the role of STAP-1 in CML pathogenesis, we infected human CML CD34^+^ cells obtained from six patients with control or STAP-1 short hairpin RNA (shRNA) lentivirus, and evaluated the ability of colony formation (Supplementary Fig. S1). It was observed that STAP-1 protein level was reduced to about 50% in CML specimens without antibiotic selection of knockdown cells. Despite of the low transduction efficiency, STAP-1 knockdown reduced recovered CML colony-forming cells to 89.86 ± 6.41% of control (Fig. 1c; *p* < 0.005). These results alluded to the possibility that the aberrant expression of STAP-1 in LSCs is involved in leukemogenesis of CML and specific features of CML stem cells.

### STAP-1 maintains LSCs in CML mice

**Fig. 2:**
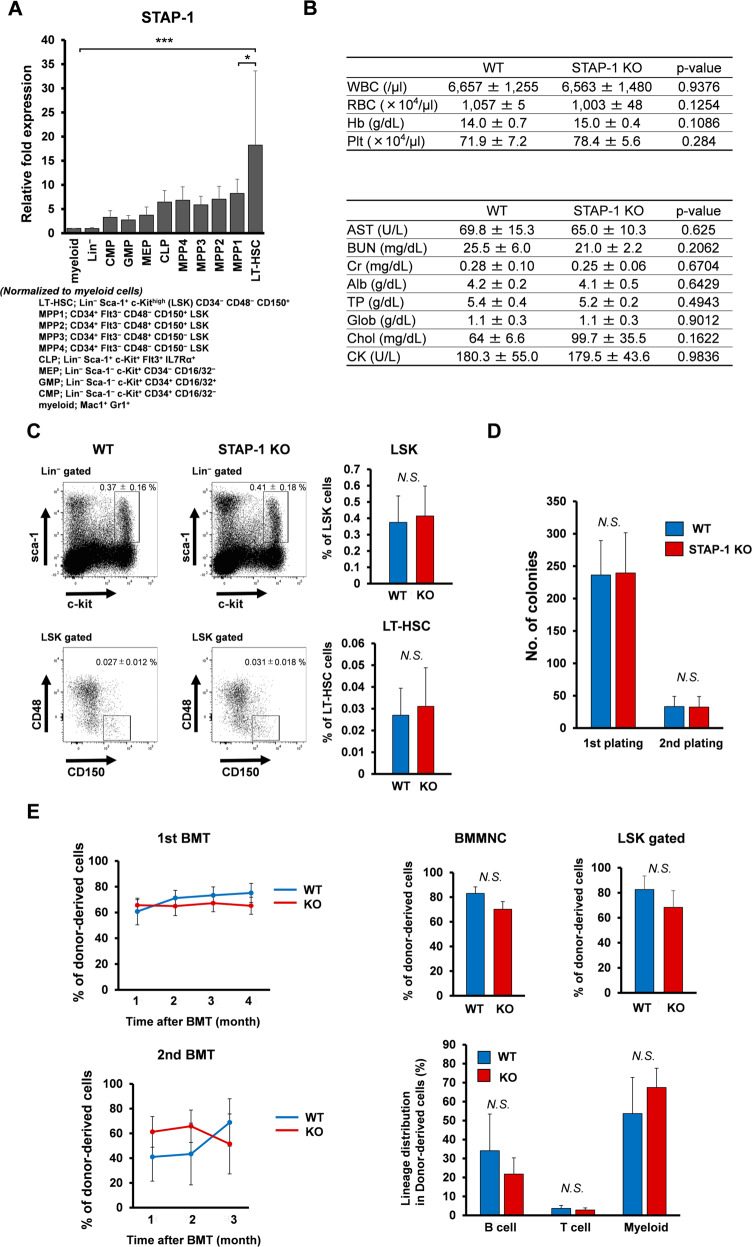
STAP-1 does not affect normal murine hematopoiesis. **a** The indicated subsets of hematopoietic cells derived from WT C57BL/6 mice were analyzed for STAP-1 expression by real-time RT-PCR (*n* = 3). STAP-1 transcript levels were normalized to myeloid subset. **b** Blood counts (WT, *n* = 5; STAP-1 KO, *n* = 7) and biochemical parameters (WT, *n* = 4; STAP-1, KO *n* = 4) were evaluated using peripheral blood. **c** The frequency of LSK and LT-HSC (CD48^−^ CD150^+^ LSK) cells from WT and STAP-1 KO mice was analyzed. Representative flow cytometry plots for identification of LT-HSC were shown (left panels). Bar graph represents mean proportion of LSK cells or LT-HSCs (WT, *n* = 9; STAP-1 KO, *n* = 8). **d** LSK cells from WT or STAP-1 KO BM (*n* = 6) were cultured in methylcellulose medium for 7 days, colonies were counted, and cells were serially replated. **e** WT or STAP-1 KO LSK cells (5000 cells, Ly5.2) were transplanted into lethally irradiated recipients (Ly5.1). At 4 months after transplantation, 2 × 10^6^ whole BM cells from the primary recipient mice were transplanted into the secondary recipient mice (Ly5.1). The left line graphs represent the mean percentages of donor chimerism in recipient’s PB at the indicated time points after transplantation (WT, *n* = 9; STAP-1 KO, *n* = 7 for the first BMT, and *n* = 11 per group for the second BMT). Upper bar graphs represent chimerism of donor-derived cells in BMMNC cells and LSK cells from primary BMT recipients of WT or STAP-1 KO LSK cells 4 months after primary BMT (WT, *n* = 9; STAP-1 KO, *n* = 7). Lower bar graph represents differentiation status (B220^+^ B cells, CD3^+^ T cells, or Mac-1/Gr-1^+^ myeloid cells) in BM cells from primary BMT recipients of WT or STAP-1 KO LSK cells 4 months after primary BMT (WT, *n* = 9; STAP-1 KO, *n* = 7). Data shown represent the mean ± SD. *p < 0.05 and ***p < 0.001 indicate significant difference. BMMNC bone marrow mononuclear cell, NS not significant.

The effect of STAP-1 on hematopoiesis remains unknown, while STAP-2 does not affect hematopoiesis at steady state [34]. Quantitative PCR experiments showed that STAP-1 mRNA level in mice is upregulated in the long-term HSC compartment (LSK CD34^−^ CD48^−^ CD150^+^). STAP-1 expression decreased according to differentiation stage (Fig. 2a). To identify the role of STAP-1 in hematopoietic cells, we generated STAP-1 knockout (KO) mice. Offsprings were born at the expected Mendelian ratio from intercrosses of heterozygotes as well as incrosses of homozygotes. Adult STAP-1 KO mice appeared to be viable and showed no apparent abnormalities. The PB cell count and biochemical parameters related to liver or kidney functions were generally normal in STAP-1 KO mice (Fig. 2b). WT and STAP-1 KO BM contained similar frequencies of multipotent stem and progenitors (Fig. 2c). With respect to the effects on HSC stemness, the numbers and sizes of colonies recovered from WT and KO Lin^−^ Sca-1^+^ c-Kit^high^ (LSK) cells were comparable in colony-forming assays (Fig. 2d). The capacity of reconstitution of KO HSCs was sustained in primary and secondary recipients of serial transplantation experiments (Fig. 2e). The BM chimerism and lineage composition were also equivalent between the two groups. These data showed that STAP-1 does not affect normal hematopoiesis.

**Fig. 3:**
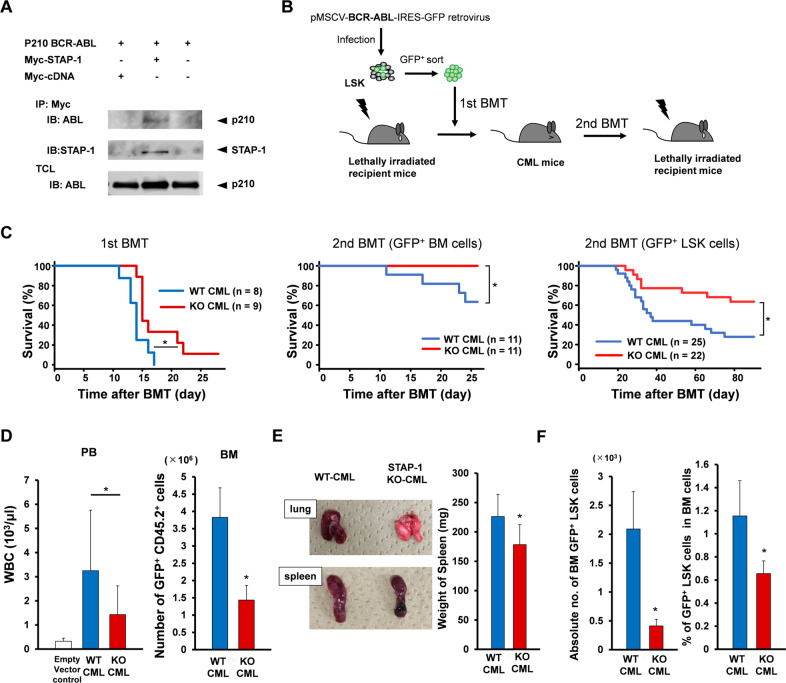
STAP-1 deficiency improves OS in in vivo CML mouse model. **a** BCR-ABL-transduced 293T cells were either or not co-transfected with Myc-tagged STAP-1. The cells were lysed, immunoprecipitated with anti-Myc antibody, and blotted with anti-ABL (upper panel) or anti-STAP-1 antibody (middle panel). An aliquot of total cell lysate (TCL) was also blotted with anti-ABL antibody (lower panel). **b** Experimental strategy for generation of CML mouse model. BCR-ABL-transduced LSK cells (1 × 10^4^ cells, Ly5.2) were transplanted into lethally irradiated recipients (Ly5.1). For serial transplantation, GFP^+^ BM cells or GFP^+^ CD45.2^+^ LSK cells from primary CML mice were isolated at day 11 after first BMT, and transplanted into lethally irradiated secondary recipient mice (Ly5.1). **c** Kaplan–Meier survival curves for primary and secondary recipients of BCR-ABL-transduced cells from WT or STAP-1 KO donor mice are shown. **d** The total numbers of peripheral white blood cells and GFP^+^ CD45.2^+^ cells in BM were analyzed at day 11 after first BMT (WT, *n* = 13; STAP-1 KO, *n* = 11). **e** Gross appearance of the lungs and spleens at day 11 after first BMT is shown. Bar graph represents the spleen weight of primary CML mice (WT, *n* = 11; STAP-1 KO, *n* = 12). **f** Leukemic stem cells (LSCs; GFP^+^ LSK cells) in BM of primary CML mice were analyzed at day 11 after first BMT. The proportions and total numbers of CML LSCs in BM were calculated (WT, *n* = 13; STAP-1 KO, *n* = 11). Data are shown as mean ± SD. *p < 0.05 indicates significant difference.

We previously demonstrated that STAP-2 interacts with BCR-ABL by its SH2-like domain [32]. Similarly, co-immunoprecipitation assays revealed that STAP-1 also binds with BCR-ABL (Fig. 3a). We examined the functional role of STAP-1 and STAP-2 in vivo using CML mouse model. BM LSK cells were transduced with p210 BCR-ABL, and transplanted into lethally irradiated recipients (Fig. 3b). As previously reported, the recipients developed and succumbed to CML within 3 weeks after the transplantation. We found that STAP-1 KO CML mice showed significantly longer survival than WT CML mice (Fig. 3c). Consistent with this finding, at day 11 after transplantation, white blood cell counts in PB and the number of leukemic cells in BM of STAP-1 KO CML mice remained significantly decreased than those of WT CML mice (Fig. 3d). In addition, STAP-1 KO CML mice displayed much less severe splenomegaly and lung hemorrhages (Fig. 3e). When LSCs in primary CML mice were analyzed, we found that the proportions and absolute numbers of GFP^+^ LSK cells in STAP-1 KO CML mice were significantly lower compared to those in WT (Fig. 3f). To evaluate the number of functional LSCs, we conducted serial transplantation (Fig. 3c). In the secondary BM transplantation (BMT), no mice injected with STAP-1 KO GFP^+^ cells developed CML, while 36.7% of the mice transplanted with GFP^+^ cells derived from WT mice developed leukemia within 28 days. Furthermore, when we used GFP^+^ LSK cells as a source of secondary BMT, STAP-1 KO CML mice significantly survived longer than WT CML mice. These results indicated that the deletion of STAP-1 decreases the number and frequency of LSCs in CML model mice.

When we evaluated the effects of STAP-2 deficiency in CML mouse model, we could not detect any differences in survival period between WT and STAP-2 KO CML mice (Supplementary Fig. S2). To explore a potential complementary effect of STAP-1 and STAP-2, STAP-1 and STAP-2 double KO CML mice were generated. Overall survival of double KO CML mice was similar to that of STAP-1 KO mice (Supplementary Fig. S2). Taken together, we concluded that STAP-1 plays a crucial role in survival of CML mice via the maintenance of LSCs.

### STAP-1 suppresses apoptosis in CML LSCs

**Fig. 4:**
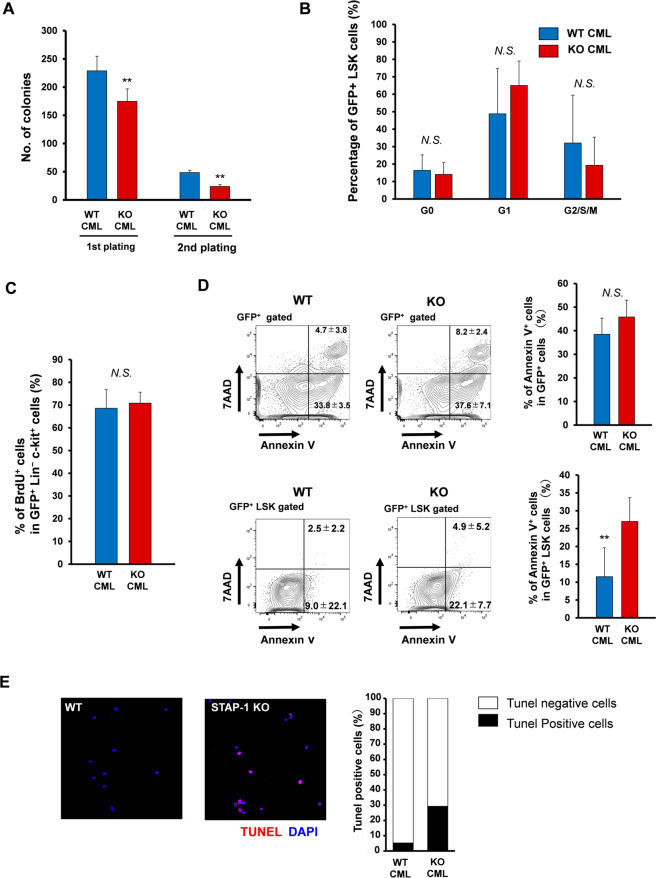
STAP-1 regulates apoptosis in CML LSCs. LSCs (GFP^+^ LSK cells) in BM of primary WT or STAP-1 KO CML mice were analyzed at day 11 after first BMT. **a** Sorted LSCs were cultured in methylcellulose medium for 7 days, colonies were counted, and cells were serially replated (*n* = 10 per group). **b** The cell cycle status of LSCs was analyzed by flow cytometry using Ki67 staining (*n* = 7 per group). **c** BrdU was administered to WT or STAP-1 KO CML mice in in vivo as described in Material and Methods to assess the cell cycle status of GFP^+^ Lin^−^ c-Kit^+^ cells with flow cytometry (*n* = 7 per group). **d** Cell apoptosis was analyzed by Annexin V and 7AAD in GFP^+^ cells or GFP^+^ LSK cells from primary WT or STAP-1 KO CML mice using flow cytometry. Representative flow cytometric data of the apoptosis are shown in left panel. The numbers in the plot indicate mean percentages ± SD of gated cells. Bar graph represents mean proportion of Annexin V^+^ cells (*n* = 7 per group). **e** Cell apoptosis was analyzed by TUNEL staining. Sorted LSCs were fixed and stained with TUNEL (red) and DAPI (blue). Bar graphs represent the proportion of TUNEL-positive cells. Data are representative of one of the two independent experiments with similar results. **p < 0.01 indicates significant difference. NS not significant.

We next performed colony-forming assay to examine whether STAP-1 affects self-renewal of CML LSCs. STAP-1 KO LSCs generated fewer colonies compared to WT LSCs during the first and second plating (Fig. 4a). We also conducted the same experiments with LK progenitor cells, and the total number and types of colonies recovered are indistinguishable between WT and STAP-1 KO (Supplementary Fig. S3), indicating that STAP-1 does not affect the progenitor fraction. When we analyzed the cell cycle status, no significant difference was observed between WT and STAP-1 KO LSCs (Fig. 4b, c). In contrast, STAP-1 KO LSCs exhibited a significantly higher apoptotic rate than WT LSCs as revealed by flow cytometric analysis (11.52 ± 8.11% in WT vs. 27.02 ± 6.66% in KO; *p* < 0.01) (Fig. 4d). The suppressive effect of STAP-1 on LSC apoptosis was confirmed with TUNEL analyses (Fig. 4e). These findings suggested that suppression of apoptosis induced by STAP-1 mediates longer survival of CML LSCs.

### STAP-1 regulates LSC apoptosis via STAT5 signaling pathway

**Fig. 5:**
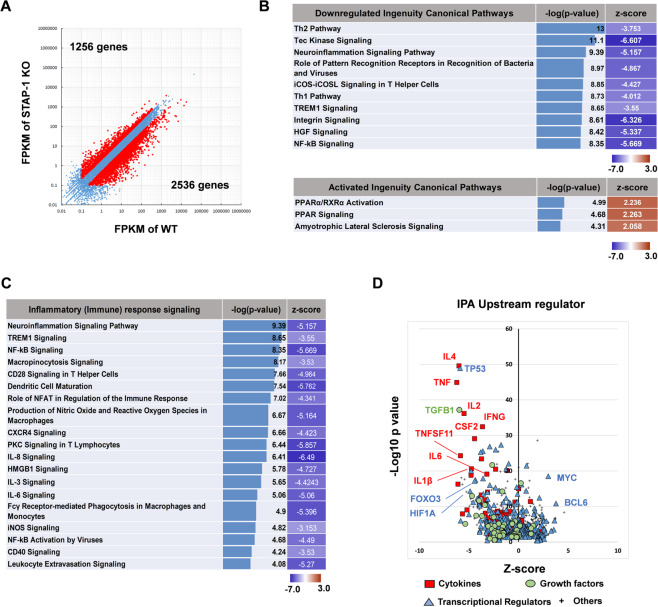
STAP-1 affects JAK/STAT signaling as well as PPAR signaling pathways. Gene expression profiles of CML LSCs at day 11 after first BMT between WT and STAP-1 KO mice were compared by RNA-seq analysis. **a** Scatter plot represents the mean of normalized counts. In all, 3792 genes (2536 downregulated genes; 1256 upregulated genes) were differentially expressed (twofold change) in STAP-1 KO LSCs (*y*-axis) relative to WT LSCs (*x*-axis). **b**–**d** Gene expression data were subjected to bioinformatics analysis by Ingenuity Pathway Analysis. For each signaling pathway, an enrichment *p* value and a *z*-score of activation were calculated. Pathways that were predicted to be downregulated or upregulated in STAP-1 KO CML LSCs compared with WT CML LSCs are shown in (**b**). Pathways involved in inflammatory responses are shown in (**c**). Upstream regulatory candidates are shown in (**d**). Candidates of interest are labeled.

To further elucidate the effects of STAP-1, we performed a gene expression analysis of WT and STAP-1 KO CML LSCs. Putative differentially expressed genes were identified by RNA-sequencing (RNA-seq; twofold change cut-off). There was a total of 3792 differentially expressed genes between WT and STAP-1 KO LSCs, among which 1256 (33.1%) genes were upregulated, while 2536 (66.9%) genes were downregulated (Fig. 5a).

Using the differentially expressed gene list, we identified the pathways that were significantly affected (*p* < 0.0001) in STAP-1 KO CML LSCs using Ingenuity Pathway Analysis. We applied *z*-score value ≥ 2.0 for activated pathways and *z*-score ≤ −3.0 for inhibited pathways. A total of 76 pathways were significantly altered in STAP-1 KO CML LSCs (Fig. 5b). The peroxisome proliferator-activated receptor (PPAR) pathway and several inflammatory pathways, such as NF-κB and JAK/STAT, which are known to be important in CML LSC maintenance [19–25], are also affected by STAP-1 ablation (Fig. 5b, c). Among upstream regulators, a number of cytokines and transcription factors, such as TGFB1, IL1B, IL6, MYC, TP53, BCL6, FOXO3, and HIF1A, were identified (Fig. 5d). These molecules have previously been reported to regulate the survival and maintenance of CML LSCs [16, 23–25, 35–37]. These results suggested that STAP-1 regulates LSC survival via various pathways.

**Fig. 6:**
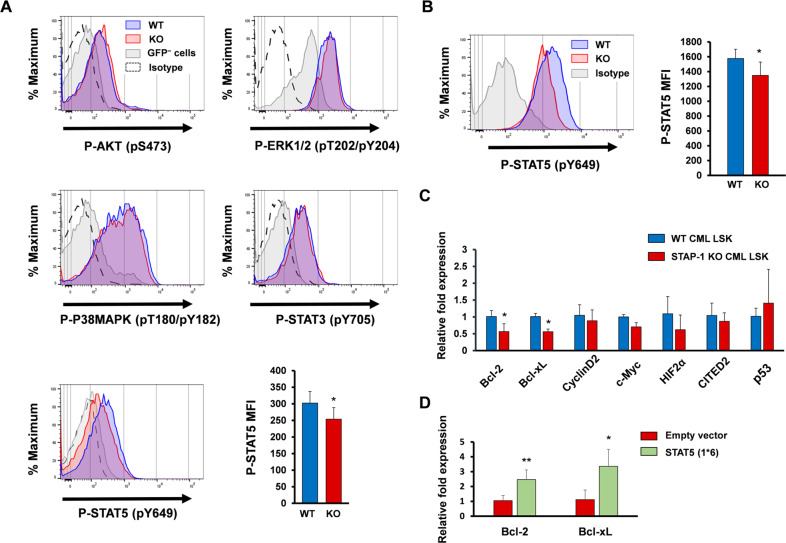
STAP-1 regulates the expression of antiapoptotic proteins via STAT5 signaling pathway. **a** Phosphorylation status of AKT (pS473), ERK1/2 (pT202/pY204), P38MAPK (pT180/pY182), STAT3 (pY705), and STAT5 (pY649) in GFP^+^ cells from WT or STAP-1 KO CML mice, at day 11 after first BMT, were analyzed by flow cytometry. Histograms show the mean fluorescence intensity (MFI) on the *x*-axis and events normalized to Mode on the *y*-axis. Histograms are representative of three independent experiments. Bar graph represents MFI of phosphorylated STAT5 (*n* = 7 per group). **b** Phosphorylation status of STAT5 (pY649) in LSCs from WT or STAP-1 KO CML mice was analyzed by flow cytometry. Histograms are representative of three independent experiments (left panel). Bar graph represents MFI of phosphorylated STAT5 (*n* = 8 per group). **c** mRNA expression of STAT5 target genes was measured by real-time RT-PCR in CML LSCs from primary CML mice (*n* = 7 per group). Results are normalized to WT mice. **d** STAP-1 KO CML LSCs were transduced with constitutively active STAT5A, and mRNA expression of Bcl-2 and Bcl-xL was measured by real-time RT-PCR (*n* = 3). Results are normalized to control. Data are shown as mean ± SD. *p < 0.05 and **p < 0.01 indicate significant difference.

Results of the transcriptome analyses led us to investigate inflammatory signaling pathways, such as JAK/STAT, PI3K/AKT, and MAPK. The evaluation of phosphorylated forms of their target proteins revealed a decrease in the levels of STAT5 in STAP-1 KO leukemic cells (Fig. 6a). The augmentation of STAT5 phosphorylation by STAP-1 deficiency was observed prominently in LSCs subset (Fig. 6b). We also investigated the mRNA expression of transcriptional targets of STAT5. As shown in Fig. 6c, the expression levels of Bcl-2 and Bcl-xL were significantly downregulated in STAP-1 KO LSCs as compared with control cells. Constitutively active STAT5 rescued the expression of Bcl-2 and Bcl-xL in LSCs inhibited by STAP-1 deletion (Fig. 6d). These data indicated that STAP-1 positively regulates the expression of antiapoptotic genes via STAT5 signaling pathway.

### STAP-1 inhibition is a possible therapeutic target in LSC elimination

**Fig. 7:**
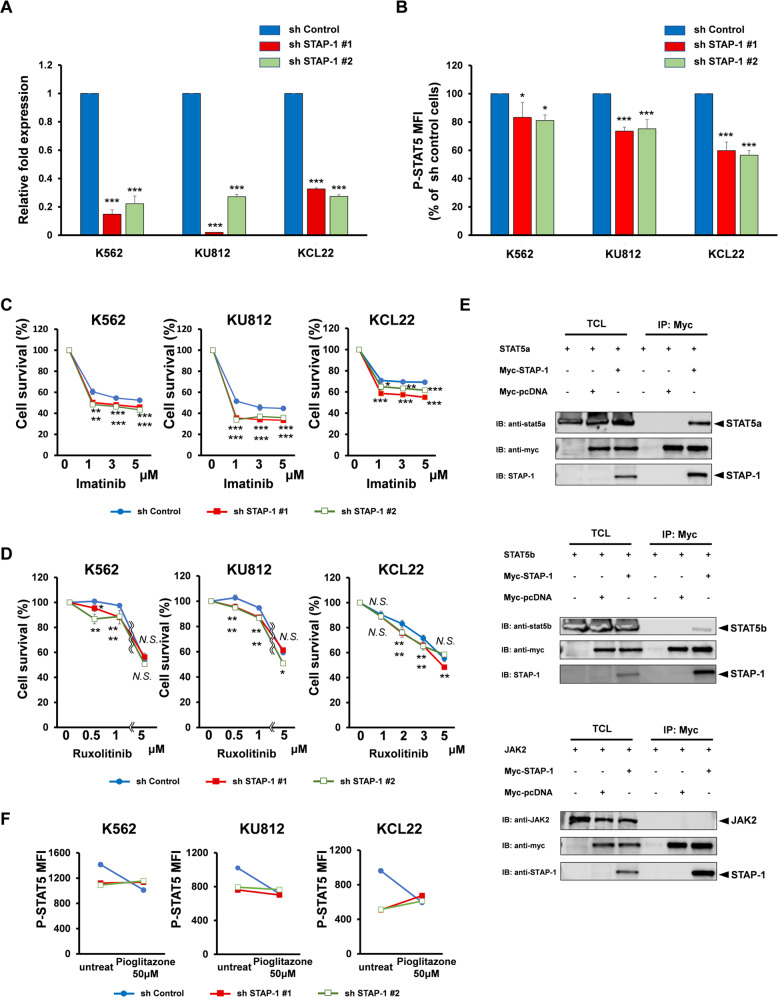
STAP-1 regulates STAT5 via JAK and PPAR signaling in CML. **a** STAP-1 knockdown CML cell lines were established using short hairpin RNA. Total RNA samples isolated from these cells were quantified by real-time RT-PCR. Data represent the levels of these mRNAs normalized to control (sh control). Data of a representative experiment, which was repeated at least three times with similar results, are shown. **b** Phosphorylation status of STAT5 (pY649) in CML cell lines was analyzed by flow cytometry. Bar graph represents MFI of phosphorylated STAT5 normalized to sh control cells (*n* = 6). CML cell lines were treated with imatinib (**c**) or ruxolitinib (**d**) at the indicated concentrations for 24 h. Growth inhibitory effect on these cells was determined. The data represent the means of triplicate experiments. Similar results were obtained for three independent experiments. **e** 293T cells were transfected with STAT5a, STAT5b, or JAK2 in either presence or absence of Myc-tagged STAP-1. The cells were lysed and immunoprecipitated with anti-Myc antibody. An aliquot of total cell lysate (TCL) and the immunoprecipitates (IP) were blotted with anti-STAT5a, anti-STAT5b, anti-JAK2, anti-Myc, or anti-STAP-1 antibody. **f** STAP-1 knockdown CML cell lines were either or not treated with pioglitazone at 50 μM for 96 h and phosphorylation status of STAT5 (pY649) was analyzed. Data of a representative experiment, which was repeated at least three times with similar results, are shown. Data represent the mean ± SD. *p < 0.05, **p < 0.01, and ***p < 0.001 indicate significant difference.

To examine the clinical significance of STAP-1 inhibition, the synergetic effects between STAP-1 deletion and TKI were studied in CML cell lines. We established STAP-1 knockdown human CML cell lines (K562, KU812, and KCL22) using shRNA. The interference of STAP-1 expression was confirmed using quantitative real-time PCR (Fig. 7a). Although knockdown of STAP-1 showed no effects on proliferation in CML cell lines, a decrease in levels of phosphorylated STAT5 was observed (Fig. 7b). When we added the first generation TKI, imatinib, into cell cultures, STAP-1 knockdown clones exhibited higher sensitivity to imatinib, indicating that STAP-1 has an additive effect with imatinib (Fig. 7c).

We next examined the effects of STAP-1 in cultures treated with JAK2 inhibitor, ruxolitinib, since JAK2-STAT5 pathway has been reported to be involved in development of CML LSC [19–21, 38]. We found that the proliferation of STAP-1 KD clones was suppressed at lower concentration than control in the presence of ruxolitinib (Fig. 7d). We explored the interaction of STAP-1 with JAK2 or STAT5. Although the direct association between STAP-1 and JAK2 was not observed, the co-immunoprecipitation experiments revealed that STAP-1 binds not only to BCR-ABL, but also with STAT5, indicating that various pathways are involved in the STAT5 regulation by STAP-1 (Fig. 7e).

Efficient eradication of LSC by the inhibition of BCR-ABL signaling using PPARγ agonist, pioglitazone, has been previously reported [25]. The treatment with pioglitazone has been shown to induce LSC death by decreasing STAT5 transcription levels. STAP-1 knockdown CML cell lines showed diminished STAT5 phosphorylation compared to control cells; however, addition of pioglitazone failed to further suppress STAT5 phosphorylation (Fig. 7f) in STAP-1 knockdown clones. These results from cultures and transcriptome analyses (Fig. 5b) indicated that PPAR signaling pathway suppresses phosphorylation status of STAT5 via STAP-1.

**Fig. 8:**
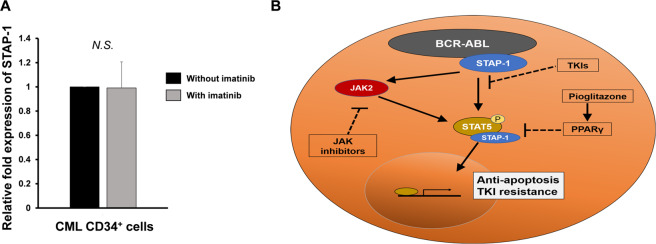
STAP-1 is a possible therapeutic target in CML. **a** Lin^−^ CD34^+^ BM cells were collected from newly diagnosed chronic CML patients (*n* = 6), and cultured in triplicates in liquid medium with or without 1 μM imatinib for 24 h. Total RNA samples isolated from these cells were quantified by real-time RT-PCR (*n* = 6 per group). Results are normalized to the untreated cells. Data are shown as mean ± SD. NS not significant. **b** Model for the role of STAP-1 in CML LSCs. STAP-1 binds to BCR-ABL and STAT5. STAP-1 positively regulates STAT5 phosphorylation status followed by change in cellular apoptosis and drug sensitivity in CML.

Using patient samples, we evaluated the effects of TKI treatment on the expression of STAP-1. STAP-1 expression was upregulated in CML LSCs as shown in Fig. 1, and it was maintained after treatment with imatinib (Fig. 8a). In addition, colony formation assay from CD34^+^ CML cells showed that the deletion of STAP-1 had the additive effects with imatinib in two out of six patients (Supplementary Fig. S4). These results suggested that STAP-1 might be involved in the resistance of imatinib in human CML LSCs.

Taken together, STAP-1 inhibition increased the sensitivity to imatinib and ruxolitinib. Furthermore, STAP-1 decreased STAT5 phosphorylation directly as well as indirectly via PPAR signaling pathway. These data indicated that STAP-1 inhibition is a possible therapeutic strategy for LSC elimination in CML.

## Discussion

In this study, we demonstrated that STAP-1 plays crucial roles in the maintenance of CML LSCs. Using CML mouse model, we showed that STAP-1 suppresses apoptosis and maintains CML LSCs (Fig. 4). STAP-1 deficiency inhibits STAT5 phosphorylation, and downregulation of antiapoptotic genes, such as Bcl-2 and Bcl-xL, in CML LSCs (Fig. 6). Furthermore, several evidences indicate that STAP-1 affects the regulation of LSCs in the both BCR-ABL-dependent and -independent ways (Fig. 7). This is the first report describing the functional role of STAP-1 in CML. Our results revealed STAP-1 to be a novel regulator of CML LSCs.

Human STAP-1 was identified as a Tec-interacting protein, termed as BRDG1, in 1999 [39]. Similar to another member of STAP family, human STAP-1 binds with the kinase domain and its structure suggested the role as an adapter protein. Consequently, murine STAP-1 was identified as a c-Kit interacting protein [28]. However, there have been few reports published for almost two decades since their discovery [28, 39–42]. Stoecker et al. reported that STAP-1 expression is induced in pro-inflammatory microglia and macrophages, which contribute to neuronal apoptosis and degeneration [41]. We also found the role of STAP-1 in NKT cell-mediated autoimmune hepatitis (manuscript in preparation). Most recently, Steeghs et al. reported that high expression of STAP-1 was identified in pediatric B-cell precursor (BCP) acute lymphoblastic leukemia (ALL) [42]. Microarray results from 572 patients showed that the expression of STAP-1 is increased in about 20% of samples including those with and without BCR-ABL fusion gene. They conducted several culture experiments using STAP-1 knocked down BCP-ALL cell lines (Nalm-6 and Kasumi-2); however, no effects on cell proliferation or drug resistance were observed [42]. In our study, the expression of STAP-1 is upregulated with CML incidence, and STAP-1 is required for survival and maintenance of LSCs in CML mice. On the other hand, we could not detect the functional role of STAP-1 in differentiated fraction of CML cells.

Previously, we have demonstrated that STAP-2 enhances BCR-ABL activity [32]. STAP-2 overexpression in p210 BCR-ABL expressing Ba/F3 cells promotes proliferation and resistance to imatinib. STAP-2 also affects downstream signaling molecules, including ERK, STAT5, Bcl-xL, and Bcl-2. Although in this study involving in vivo CML mouse model, STAP-2 did not significantly affect the survival of CML mice; however, some of their phenotypes, such as the reduction in number of LSCs, were similar to STAP-1 deficient model (data not shown).

Our analysis of CML LSCs showed that STAP-1 strongly affected phosphorylation of STAT5 (Fig. 6). STAT5 has been known to be one of the most important regulators of survival and growth of CML [43–45]. Several mechanisms have been suggested to activate STAT5 in CML. As a tyrosine kinase, BCR-ABL induces phosphorylation of STAT5 [44, 45]. The direct physical interaction between BCR-ABL and STAT5 was also previously reported [43, 44]. In CML cells, it is known that JAK2 is constitutively activated, inducing several downstream molecules, including STAT5 [19, 20]. Recent studies have reported the complex of BCR-ABL and JAK2 protein as a regulator of BCR-ABL kinase activity [20]. We here demonstrated that binding of STAP-1 to BCR-ABL as well as STAT5 could enhance STAT5 signaling.

Importantly, recent studies have indicated that STAT5 activation is regulated independently by BCR-ABL in CML LSCs. For instance, JAK2 is activated following binding of hematopoietic growth factors to their receptors during CML leukemogenesis; it has been reported that the treatment with nilotinib and ruxolitinib leads to eradication of primary human CML LSCs in vitro [21], and ongoing clinical trials are attempting to improve outcomes of CML patients [38]. Prost et al. showed that PPARγ agonist, pioglitazone, can purge the residual CML LSC pool by downregulating expression of STAT5 and its downstream targets, Hif2a and Cited2, which are key regulators of the stemness of CML LSCs [25]. We showed here that PPAR signaling pathway suppresses phosphorylation status of STAT5 via STAP-1. Furthermore, STAP-1 directly binds to STAT5. STAP-1 is likely to be involved in multiple cascades leading to STAT5 activation. The underlying mechanisms should be elucidated in further studies.

From the clinical point of view, our report suggested that STAP-1 inhibition could be a novel therapeutic measure for CML that help to overcome resistance and disease persistence. The putative mechanism of resistance might be involved conformational changes of BCR-ABL, which is led to be bind with STAP-1. Interestingly, our study also implied that BCR-ABL-independent STAT5 inhibition via STAP-1 signaling could be useful for LSC elimination. TKI resistance depends on the transcriptional activity of STAT5, since the STAT5 target genes, such as Bcl-2 and Bcl-xL, inhibit apoptosis and cytotoxicity [46]. It has been reported that activation of STAT5 affects cell cycle, apoptosis, and upregulation of ROS, which contribute to an increased mutation rate and resistance to TKI [46, 47]. Although several clinical trials, involving treatment with ruxolitinib or pioglitazone, have currently been running, the eradication of LSCs remains a major challenge for CML treatment. Considering the effects of STAP-1 on normal hematopoiesis, STAP-1 could represent an attractive therapeutic target for CML treatment by inhibiting STAT5 activity in CML LSCs.

In summary, this work demonstrated critical roles of STAP-1 in the maintenance of CML LSCs. STAP-1 positively regulates CML LSCs via suppression of the apoptosis through the regulation of STAT5. These findings provided a potential molecular mechanism for the pathogenesis of CML. Our data supported the evidence that STAP-1 could act as a novel therapeutic target for CML via elimination of CML LSCs.

## Materials and methods

### Human samples

BM samples from normal volunteers and patients with CML-CP at diagnosis were obtained after receiving written informed consent in accordance with the Declaration of Helsinki. This study protocol was approved by the institutional review board of Osaka University Hospital.

### Mice

STAP-1 KO mice on C57BL/6J background (Ly5.2) were generated with a targeted disruption in the STAP-1 gene locus [29]. The congenic C57BL/6J strain (C57BL/6SJL; Ly5.1) was obtained from Jackson laboratory and used for transplantation experiments. Overall, 8–14-week-old mice were used in all experiments. All experimental procedures were conducted under protocols approved by Institutional Animal Care and Use Committee of Osaka University.

### Flow cytometric analysis

Flow cytometric analysis and sorting were performed using standard multicolor immunofluorescent staining protocols with a FACS Aria IIu (BD Biosciences). The indicated antibodies used for flow cytometric sorting and analysis were listed in Supplementary Table 1. A mixture of monoclonal antibodies recognizing CD3e, CD8, CD11b/Mac1, CD19, B220, Gr-1, and Ter119 was used to identify murine lineage cells. A mixture of monoclonal antibodies recognizing CD3, CD14, CD16, CD19, CD20, CD56, and CD235a was used to identify human lineage cells. FlowJo software (Tree Star) was used for data analysis. For intracellular assays, cells were fixed and permeabilized using Cytofix/Cytoperm buffer (BD Bioscience) and stained with the indicated antibodies.

### Bone marrow transplantation

In all, 5000 (LSK) cells sorted with FACS Aria were transplanted into lethally irradiated C57BL/6SJL mice, together with 5 × 10^5^ BM cells from C57BL/6SJL mice. Four months after the first transplantation, serial transplantation was performed by transferring 2 × 10^6^ BM cells from primary recipients.

### Preparation of retroviral particles

The cDNA encoding human BCR-ABL was cloned into the EcoRI site of the pMSCV-ires-GFP vector to generate retrovirus particles [48]. Constitutively active form of STAT5A (1*6) was subcloned into pMSCV-ires-GFP vector. Each retroviral vector was transfected into 293T cells by polyethylenimine. After 48 h the culture supernatant was collected, concentrated 100-fold in volume, and filtered.

### Generation of CML model mice

The transduction of BCR-ABL to LSK cells was conducted as previously described [49]. After infection, sorted GFP positive cells (1 × 10^4^) were transplanted intravenously into lethally irradiated C57BL/6SJL mice together with 3 × 10^5^ BM cells from C57BL/6SJL mice. For serial transplantation, 1 × 10^6^ GFP^+^ BM cells or 5 × 10^4^ GFP^+^ LSK cells were isolated from recipient mice 11 days after the first transplantation, and transplanted into lethally irradiated recipient mice together with BM cells from C57BL/6SJL mice.

### Methylcellulose colony formation assay

Cells were cultured in MethoCult medium (GF M3434 and H4434, Stem Cell Technologies). The numbers of colonies were counted after 7 days. For serial replating assays, cells were harvested from the first plates and replated in fresh methylcellulose medium. The numbers of colonies were counted again after 7 days.

### Cell cycle analysis

To determine the cell cycle status in vivo, mice were administered BrdU intraperitoneally (100 mg/kg of body weight in saline) 12 h before analysis. Numbers of BrdU^+^ cells were assessed with flow cytometry. For Ki67 staining, cells were fixed and permeabilized before staining with APC conjugated anti-Ki67 antibody.

### Apoptosis assay

Isolated GFP^+^ cells were stained using PE Annexin V apoptosis Detection Kit (BD Biosciences). For TUNEL staining, GFP^+^ LSK cells were attached to glass slides by cytospin, fixed with 4% paraformaldehyde, and stained according to the manufacturer’s protocols (Sigma-Aldrich).

### RNA-sequencing analysis

GFP^+^ CD45.2^+^ LSK cells from WT and STAP-1 KO CML mice were sorted by FACS, and total RNA was extracted with an RNeasy Mini Kit (Qiagen, Valencia, CA) according to the manufacturer’s instructions. Each cDNA was generated using a Clontech SMART-Seq v4 Ultra^®^ Low Input RNA Kit for Sequencing (Takara Clontech). Each library was prepared using a Nextera XT DNA Library Prep Kit (Illumina) according to the manufacturer’s instructions. Whole transcriptome sequencing was applied to the RNA samples with use of on an Illumina HiSeq 2500 platform in a 75-base single-end mode. Sequenced reads were mapped to the mouse reference genome sequences (mm10) using TopHat ver. 2.0.13 in combination with Bowtie2 ver. 2.2.3 and SAMtools ver. 1.0. The number of fragments per kilobase of exon per million mapped fragments was calculated using Cuffnorm ver. 2.2.1. Access to raw data concerning this study was submitted under Gene Expression Omnibus accession number GSE127984. The data sets were analyzed using Ingenuity Pathway Analysis (Ingenuity Systems Inc).

### Quantitative real-time RT-PCR analysis

Total RNA was isolated from GFP^+^ CD45.2^+^ LSK cells from CML mice using the RNeasy Mini kit and converted to cDNA by High-Capacity cDNA Reverse Transcription kit (Applied Biosystems). Real-time quantitative PCR was performed using Fast SYBR Green Master Mix (Applied Biosystems) and Taqman gene-specific primers. Primers and probes are reported in Supplementary Table 2.

### Cell cultures

Human CML cell lines (K562, KU812, and KCL22) were obtained from the JCRB Cell Bank (JCRB0019, JCRB0104, JCRB1317), and maintained in RPMI (nacalai tesque) supplemented with 10% fetal bovine serum. For cell proliferation and viability assay, the Cell Counting Kit-8 (Sigma-Aldrich) was used as recommended by the manufacturer. In brief, the cells (1–2 × 10^4^/well) in 96-well plates were cultured for the indicated periods. CCK-8 reagent was added and incubated another 4 h at 37 °C and Optical Density was measured at 450 nm with a microplate reader. In some experiments, imatinib mesylate, pioglitazone (Sigma-Aldrich), or ruxolitinib (ChemScene) was added in the cultures.

### Lentivirus transduction in human CML CD34+ cells

Lentiviruses were produced by transfection in 293T with MISSION shRNA vectors (Sigma-Aldrich). CML CD34^+^ cells (4 × 10^6^ cells/ml) were infected by spinoculation (800 g, 90 min, 30 °C). Two adjuvants, poloxamer 407 (100 μg/mL, Sigma-Aldrich) and prostaglandin E2 (10 μM, Merck), were added to the culture medium [50]. Cells were harvested 72 h later, and the knockdown effect was examined by western blot analysis.

### Establishment of STAP-1 knockdown CML cell lines

Stable STAP-1 knockdown CML cell lines were established by transfection with MISSION shRNA lentiviral vectors (pLKO.1-puro) targeting human STAP-1 and selection with puromycin (clone IDs: NM_012108.1-747s1c1, NM_012108.1-692s1c1). For control, we used MISSION pLKO.1-puro empty vector (#SHC001).

### Immunoprecipitation and immunoblotting assay

The cells were harvested and lysed in lysis buffer (50 mM Tris-HCl, pH 7.6, 0.15 M NaCl containing 1% Nonidet P-40, 1 μM sodium orthovanadate, 1 μM phenylmethylsulfonyl fluoride, and 10 μg/ml each aprotinin, pepstatin, and leupeptin). The immunoprecipitates from cell lysates were resolved on SDS-PAGE and transferred to Immobilon filters (Millipore). Immunoblotting was performed with appropriate antibodies. Immunoreactive proteins were visualized by an Odyssey infrared imaging system (LI-COR Biotechnology). Antibodies against c-Abl (rabbit polyclonal), Myc (mouse monoclonal; clone 9E10), STAT5a (rabbit polyclonal), STAT5b (rabbit polyclonal), and JAK2 (rabbit polyclonal) were purchased from Santa Cruz Biotechnology. Antibodies against STAP-1 (rabbit polyclonal) were purchased from Sigma-Aldrich.

### Statistical analysis

Statistical differences were determined using the Student’s *t* test. Survival curves were calculated using the Kaplan–Meier method and analyzed with the long-rank test. Statistics were analyzed using JMP Pro 13.0.0 software (SAS Institute Inc). No samples were excluded from the analysis. The data had a normal distribution, and the variance was similar between the groups being compared. No statistical method was used to predetermine sample sizes. Sample size was based on previous experience with experimental variability. The experiments were not randomized. The investigators were not blinded to allocation during experiments or outcome assessment.

## Supplementary information

Supplementary figures and tables
