# Supplementary material for: Signal-transducing adapter protein-1 is required for maintenance of leukemic stem cells in CML
Source: Oncogene. 2020 Jul 13;39(34):5601–15. doi: 10.1038/s41388-020-01387-9 (PMC7441008; doi:10.1038/s41388-020-01387-9)
Supplement: Supplementary file 1 — Supplementary figures and tables [file 41388_2020_1387_MOESM1_ESM.docx]

**Supplementary Figures, Figure Legends and Tables**


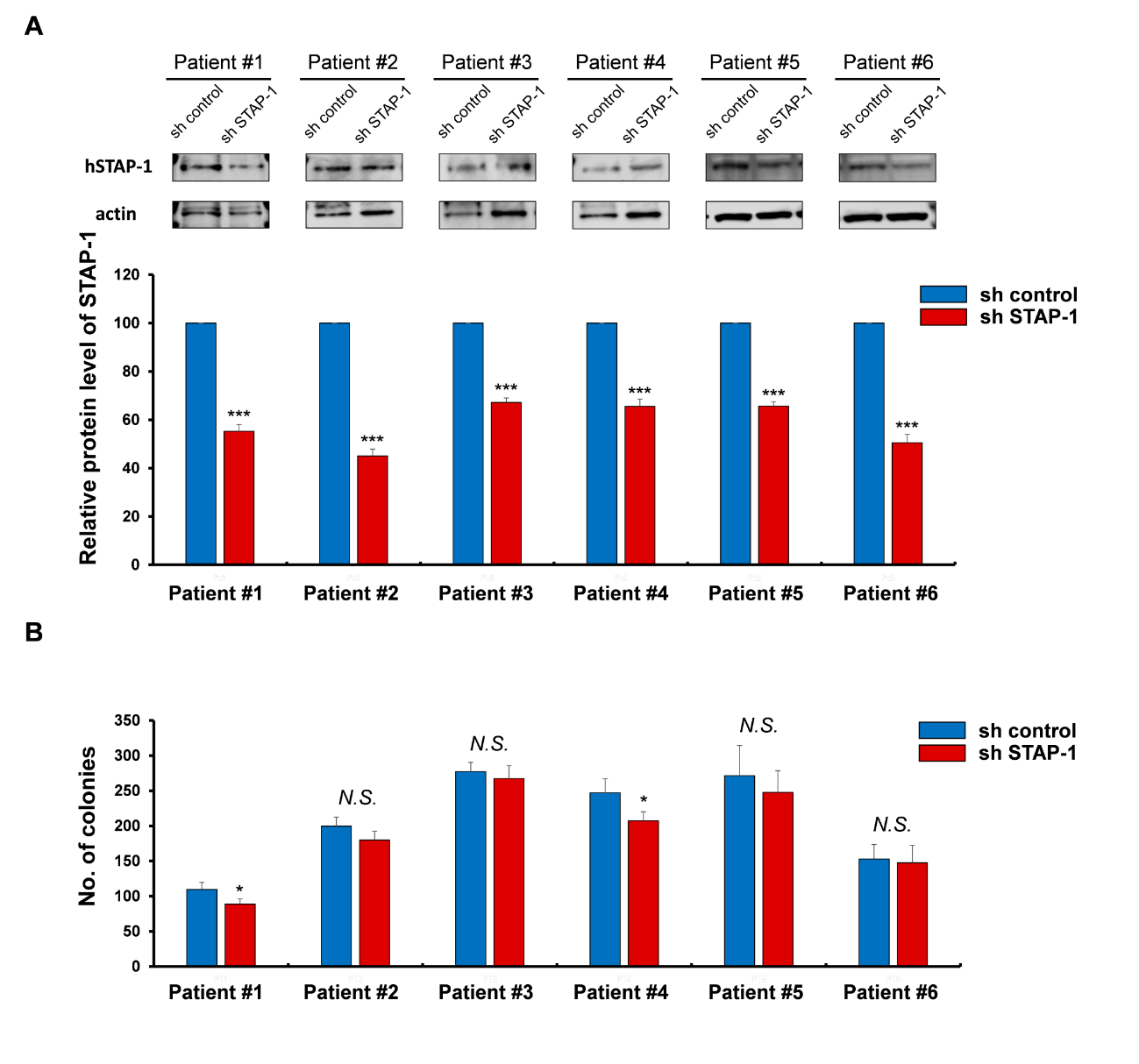


**Supplementary Figure 1. STAP-1 deletion inhibits the ability of colony formation of human CML LSCs.**

(a, b) Lin^-^ CD34^+^ BM cells from newly diagnosed chronic CML patients (n = 6) were transduced with control or STAP-1 shRNA lentivirus for 72 hours. (a) These cells were lysed and blotted with anti-STAP-1 antibody to measure the STAP-1 protein level. Data represent the levels of STAP-1 protein normalized to that of β-actin internal control and are expressed relative to the value of control shRNA samples. (b) These cells were cultured in triplicates in methylcellulose medium for 14 days, and colonies were counted. Data represent the mean ± SD. **p* < 0.05, and ****p* < 0.001 indicate significant difference. NS, not significant.


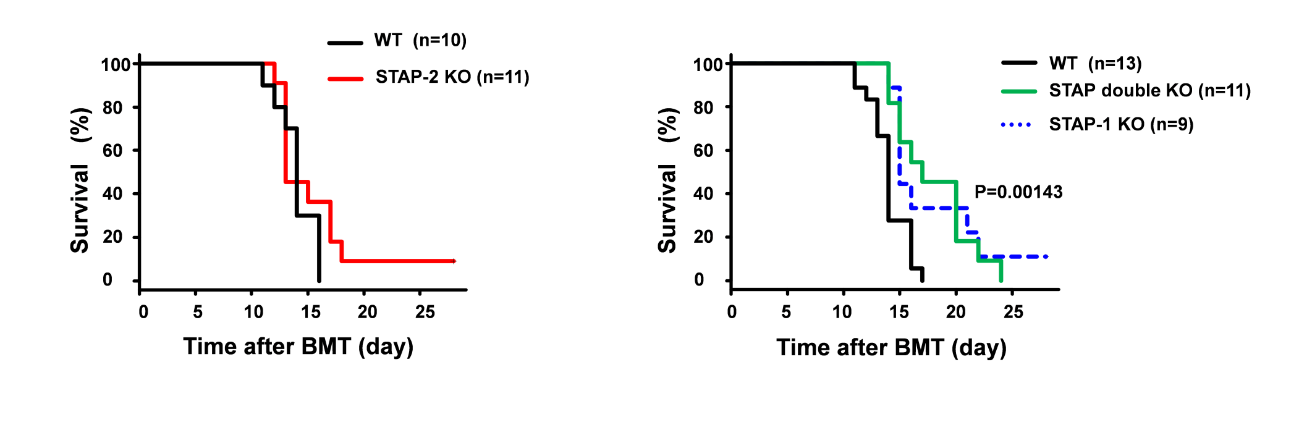


**Supplementary Figure 2.**

**STAP-2 deficiency does not affect survival of primary CML mice.**

Kaplan-Meier survival curves for primary recipients of BCR-ABL-transduced LSK cells from WT, STAP-2 KO (left graph), or STAP1/2 double KO donor mice (right graph) are shown.


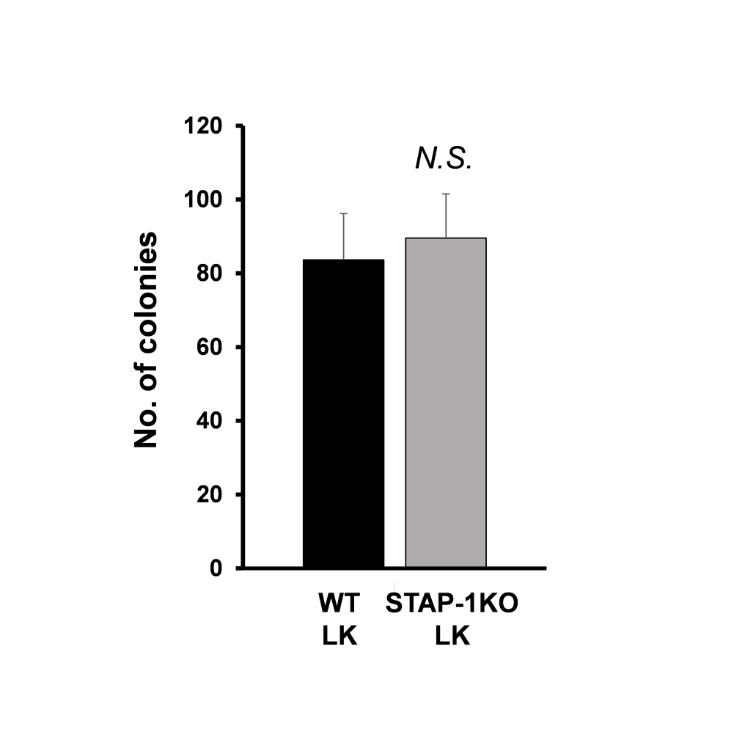


**Supplementary Figure 3.**

**STAP-1 does not affect the progenitor fraction.**

Sorted LK cells were cultured in methylcellulose medium for 7 days, colonies were counted (n = 9 per group). NS, not significant.


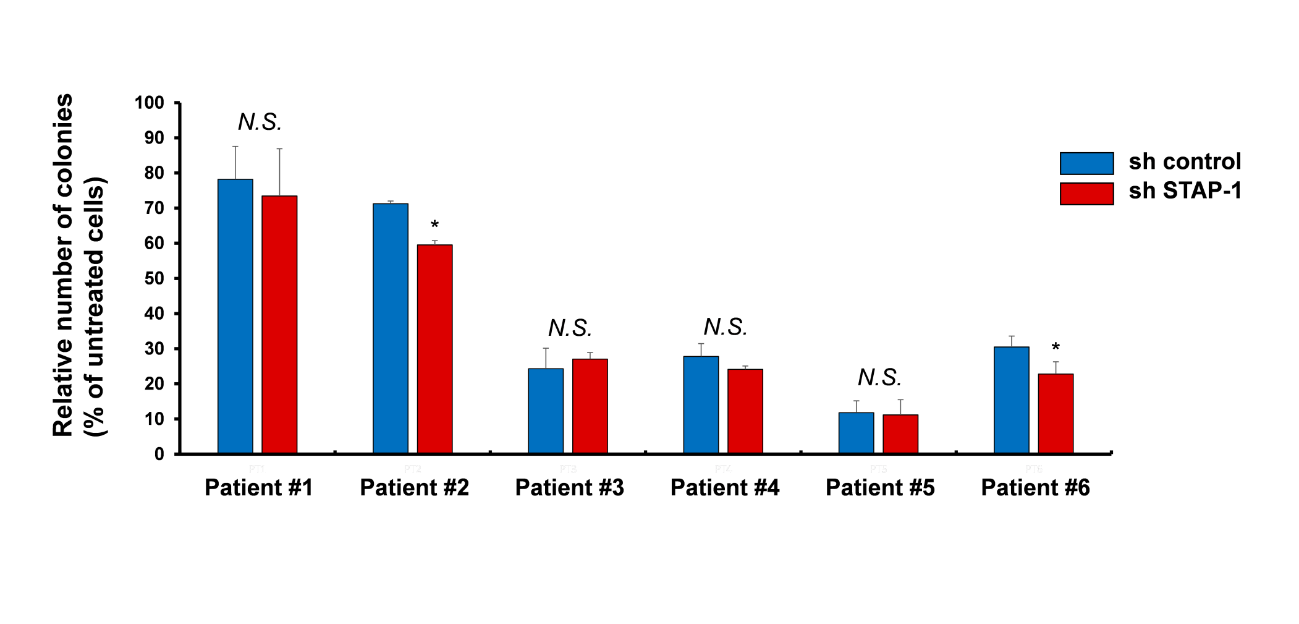
**Supplementary Figure 4. STAP-1 deletion contributes to the elimination of human CML LSCs.**

Lin^-^ CD34^+^ BM cells from newly diagnosed chronic CML patients (n = 6) were transduced with control or STAP-1 shRNA lentivirus for 72 hours. These cells were cultured in triplicates in methylcellulose medium with or without imatinib (1μM) for 14 days, and colonies were counted. Results are expressed relative to untreated cells. Data represent the mean ± SD. **p* < 0.05 indicate significant difference. NS, not significant.

**Supplementary Table 1.**

**Antibodies used for flow cytometry.**

**Supplementary Table 2.**

**List of primers and probes used in RT-qPCR.**

| **Primer and Sequence** | **Sourse** | **Identifier** |
| --- | --- | --- |
| Mouse *Bcl-2* Forward  GTCCCGCCTCTTCACCTTTCAG | FASMAC CO., LTD. | N/A |
| Mouse *Bcl-2* Reverse  GATTCTGGTGTTTCCCCGTTGG | FASMAC CO., LTD. | N/A |
| Mouse *Bcl-xL* Forward  AACATCCCAGCTTCACATAACCCC | FASMAC CO., LTD. | N/A |
| Mouse *Bcl-xL* Reverse  GCGACCCCAGTTTACTCCATCC | FASMAC CO., LTD. | N/A |
| Mouse *Cited2* Forward  TTGGACCGCATCAAGGAG | FASMAC CO., LTD. | N/A |
| Mouse *Cited2* Reverse  TTAATTCACACCGAAGAAGTTG | FASMAC CO., LTD. | N/A |
| Mouse *c-Myc* Forward  CGGACACACAACGTCTTGGAA | FASMAC CO., LTD. | N/A |
| Mouse *c-Myc* Reverse  AGGATGTAGGCGGTGGCTTTT | FASMAC CO., LTD. | N/A |
| Mouse *CyclinD2* Forward  GCGTGCAGAAGGACATCCA | FASMAC CO., LTD. | N/A |
| Mouse *CyclinD2* Reverse  CACTTTTGTTCCTCACAGACCTCTAG | FASMAC CO., LTD. | N/A |
| Mouse *Hif2α* Forward  CCTGCTGTCCTGCCTTATCATC | FASMAC CO., LTD. | N/A |
| Mouse *Hif2α* Reverse  TCAGTTCCAAGATTCTGTCGTCAC | FASMAC CO., LTD. | N/A |
| Mouse *p53* Forward  TTTTGAAGGCCCAAGTGAAG | FASMAC CO., LTD. | N/A |
| Mouse *p53* Reverse  TCTTCTGTACGGCGGTCTCT | FASMAC CO., LTD. | N/A |
| Taqman probe for human *GAPDH* (Hs02786624_g1) | Thermo Fisher Scientific | Cat# 4331182 |
| Taqman probe for human *STAP-1* (Hs00201585_m1) | Thermo Fisher Scientific | Cat# 4331182 |
| Taqman probe for human *STAP-2* (Hs00214588_m1) | Thermo Fisher Scientific | Cat# 4331182 |
